# Supplementary material for: Enhanced SVM-based model for predicting cyberspace vulnerabilities: Analyzing the role of user group dynamics and capital influx
Source: PLoS One. 2025 Jul 17;20(7):e0327476. doi: 10.1371/journal.pone.0327476 (PMC12270157; doi:10.1371/journal.pone.0327476)
Supplement: Attachment — Submitted filename: Response to reviewers .docx [file pone.0327476.s003.docx]

PONE-D-25-20493

Application of the Improved Support Vector Machine Model for Cyberspace Vulnerability Prediction Technology from the Perspective of User Group Changes Based on Capital Influx

PLOS ONE

Dear Dr. Long,

Thank you for submitting your manuscript to PLOS ONE. After careful consideration, we feel that it has merit but does not fully meet PLOS ONE’s publication criteria as it currently stands. Therefore, we invite you to submit a revised version of the manuscript that addresses the points raised during the review process.


Please submit your revised manuscript by Jul 11 2025 11:59PM. If you will need more time than this to complete your revisions, please reply to this message or contact the journal office at [plosone@plos.org](mailto:plosone@plos.org). When you're ready to submit your revision, log on to https://www.editorialmanager.com/pone/ and select the 'Submissions Needing Revision' folder to locate your manuscript file.

Please include the following items when submitting your revised manuscript:

- A rebuttal letter that responds to each point raised by the academic editor and reviewer(s). You should upload this letter as a separate file labeled 'Response to Reviewers'.
- A marked-up copy of your manuscript that highlights changes made to the original version. You should upload this as a separate file labeled 'Revised Manuscript with Track Changes'.
- An unmarked version of your revised paper without tracked changes. You should upload this as a separate file labeled 'Manuscript'.

If you would like to make changes to your financial disclosure, please include your updated statement in your cover letter. Guidelines for resubmitting your figure files are available below the reviewer comments at the end of this letter.

If applicable, we recommend that you deposit your laboratory protocols in protocols.io to enhance the reproducibility of your results. Protocols.io assigns your protocol its own identifier (DOI) so that it can be cited independently in the future. For instructions see: <https://journals.plos.org/plosone/s/submission-guidelines#loc-laboratory-protocols>. Additionally, PLOS ONE offers an option for publishing peer-reviewed Lab Protocol articles, which describe protocols hosted on protocols.io. Read more information on sharing protocols at <https://plos.org/protocols?utm_medium=editorial-email&utm_source=authorletters&utm_campaign=protocols>.

We look forward to receiving your revised manuscript.

Kind regards,

Pankaj Bhambri, Ph.D.
Academic Editor
PLOS ONE

Journal Requirements:

When submitting your revision, we need you to address these additional requirements.

1. Please ensure that your manuscript meets PLOS ONE's style requirements, including those for file naming. The PLOS ONE style templates can be found at https://journals.plos.org/plosone/s/file?id=wjVg/PLOSOne_formatting_sample_main_body.pdf and https://journals.plos.org/plosone/s/file?id=ba62/PLOSOne_formatting_sample_title_authors_affiliations.pdf

2. PLOS requires an ORCID iD for the corresponding author in Editorial Manager on papers submitted after December 6th, 2016. Please ensure that you have an ORCID iD and that it is validated in Editorial Manager. To do this, go to ‘Update my Information’ (in the upper left-hand corner of the main menu), and click on the Fetch/Validate link next to the ORCID field. This will take you to the ORCID site and allow you to create a new iD or authenticate a pre-existing iD in Editorial Manager.

3. [Please include captions for your Supporting Information files at the end of your manuscript, and update any in-text citations to match accordingly. Please see our Supporting Information guidelines for more information: http://journals.plos.org/plosone/s/supporting-information.](https://www.editorialmanager.com/pone/Please%20include%20captions%20for%20your%20Supporting%20Information%20files%20at%20the%20end%20of%20your%20manuscript,%20and%20update%20any%20in-text%20citations%20to%20match%20accordingly.%20Please%20see%20our%20Supporting%20Information%20guidelines%20for%20more%20information:%20http:/journals.plos.org/plosone/s/supporting-information.%20)

4. Please review your reference list to ensure that it is complete and correct. If you have cited papers that have been retracted, please include the rationale for doing so in the manuscript text, or remove these references and replace them with relevant current references. Any changes to the reference list should be mentioned in the rebuttal letter that accompanies your revised manuscript. If you need to cite a retracted article, indicate the article’s retracted status in the References list and also include a citation and full reference for the retraction notice.

Additional Editor Comments :

Minor Revisions are Required.


[Note: HTML markup is below. Please do not edit.]

Reviewers' comments:

Reviewer's Responses to Questions

**Comments to the Author**

1. Is the manuscript technically sound, and do the data support the conclusions?

The manuscript must describe a technically sound piece of scientific research with data that supports the conclusions. Experiments must have been conducted rigorously, with appropriate controls, replication, and sample sizes. The conclusions must be drawn appropriately based on the data presented.

Reviewer #1: Partly

Reviewer #2: Yes

Reviewer #3: Yes

Reviewer #4: Yes

2. Has the statistical analysis been performed appropriately and rigorously?

Reviewer #1: No

Reviewer #2: Yes

Reviewer #3: Yes

Reviewer #4: Yes

3. Have the authors made all data underlying the findings in their manuscript fully available?

The [PLOS Data policy](http://www.plosone.org/static/policies.action#sharing) requires authors to make all data underlying the findings described in their manuscript fully available without restriction, with rare exception (please refer to the Data Availability Statement in the manuscript PDF file). The data should be provided as part of the manuscript or its supporting information, or deposited to a public repository. For example, in addition to summary statistics, the data points behind means, medians and variance measures should be available. If there are restrictions on publicly sharing data—e.g. participant privacy or use of data from a third party—those must be specified.

Reviewer #1: Yes

Reviewer #2: Yes

Reviewer #3: Yes

Reviewer #4: Yes

4. Is the manuscript presented in an intelligible fashion and written in standard English?

PLOS ONE does not copyedit accepted manuscripts, so the language in submitted articles must be clear, correct, and unambiguous. Any typographical or grammatical errors should be corrected at revision, so please note any specific errors here.

Reviewer #1: No

Reviewer #2: Yes

Reviewer #3: Yes

Reviewer #4: Yes

5. Review Comments to the Author

Please use the space provided to explain your answers to the questions above. You may also include additional comments for the author, including concerns about dual publication, research ethics, or publication ethics. (Please upload your review as an attachment if it exceeds 20,000 characters)

Reviewer #1: The manuscript titled "Application of the Improved Support Vector Machine Model for Cyberspace Vulnerability Prediction Technology from the Perspective of User Group Changes Based on Capital Influx" presents a novel approach using a Threat-Aware Support Vector Machine (TASVM) model with adaptive kernel optimization to improve vulnerability prediction in cyberspace. The paper addresses a relevant and timely problem and demonstrates meaningful performance improvements over traditional SVM, decision tree, neural network, and random forest models.
However, several important concerns limit the manuscript’s readiness for publication in its current form:
1. Technical Soundness-
The proposed TASVM model integrates multiple dynamic kernel mechanisms to enhance adaptability to nonlinear attacks, which is a promising contribution. The use of CICIDS 2017 and UNSW-NB15 datasets is appropriate, and the experimental results are aligned with the study’s goals. However, the lack of detail in implementation (e.g., hyperparameter tuning strategies, exact kernel switching conditions, and code reproducibility) and no mention of model limitations in scalability and computation under real-time conditions reduce confidence in the system’s practical viability.
Reply: Thank you for your comments. According to your suggestions, the content of Section 3.3 has been revised to discuss the experimental implementation details of this paper, including providing the design results of model hyperparameters and discussing the scalability and computational limitations of the model under real-time conditions. As shown in Table 1, the threat-aware support vector machine model proposed in this study integrates dynamic kernel parameter adjustment, attack perception factors, and kernel switching mechanisms for cyberspace vulnerability prediction. Two datasets were selected, and after data preprocessing, the model performance was tested through cross-validation.

2. Statistical Analysis-
The manuscript does not include significance testing, confidence intervals, or variability measures (e.g., standard deviation across cross-validation folds). While average accuracy and other performance metrics are reported, the absence of statistical validation undermines the strength of the conclusions. This is a critical shortcoming that must be addressed to meet PLOS ONE's standards.
Reply: Thank you for your suggestions. According to your advice, the content of the results section in this paper has been revised, including adding the results of significance analysis and conducting an in-depth discussion to improve the quality of this paper. As shown in Table 2, the threat-aware support vector machine model proposed in this study, by integrating dynamic kernel parameter adjustment, attack perception factors, and kernel function switching mechanisms, has achieved significant improvements in cyberspace vulnerability prediction performance on the CICIDS 2017 and UNSW-NB15 datasets.

3. Language and Clarity-
Although the manuscript is generally intelligible, the writing includes numerous grammatical issues, awkward sentence constructions, and stylistic inconsistencies. These issues negatively impact readability and should be corrected through professional language editing.
Reply: Thank you for your reminder. In accordance with your reminder, the entire content of this paper has been comprehensively proofread, and the grammar and sentences have been fully optimized to comprehensively improve the readability and content quality of this paper.

4. Data Availability-
The authors appropriately use two public datasets and state that all data are available within the manuscript and supporting information, which meets the journal’s data availability requirement.
Reply: Thank you for your comments. Thank you very much for reviewing and guiding this paper. Based on your suggestions, this paper has been revised in Section 3.1 to highlight the rationale for the data by providing a more detailed explanation and discussion of the data. This study selects two publicly available cybersecurity datasets with industry representativeness: the Canadian Institute for Cybersecurity Intrusion Detection System 2017 (CICIDS 2017) released by the Canadian Institute for Cybersecurity and the Network-Based Intrusion Detection Evaluation Dataset 2015 (UNSW-NB15) developed by the University of New South Wales. The aim is to enhance the model's universality through cross-validation of multi-scenario data features.

5. Additional Suggestions-
Include significance testing (e.g., t-tests or ANOVA) to statistically validate model improvements. Add variability metrics (e.g., standard deviation, confidence intervals) for reported performance scores. Clarify how the attack-aware factor (αc) and skewness threshold (θ) are calculated and adjusted. Provide runtime benchmarks or discuss deployment feasibility for real-time systems. Improve figures for clarity, and ensure all abbreviations are defined on first use. Submit the manuscript for professional language editing.

Reply: Thank you for your suggestions. According to your advice, the content of the results section in this paper has been revised, including adding the results of significance analysis and variability measurement. Then, Section 3.3 clarifies the calculation and adjustment methods of attack perception factors and skewness thresholds. Meanwhile, some figures have been modified to improve the quality. Finally, the manuscript has been professionally edited to enhance the overall content quality. This study focuses on the impact of user group changes on cyberspace vulnerability prediction in the context of capital inflow, proposes a threat-aware support vector machine model, improves the non-linear attack recognition ability through dynamic adaptive kernel optimization, and verifies the effectiveness of the model on the dataset.

Reviewer #2: Thank you to the Author and Editor(s) for this opportunity to provide feedback.

This Original Submission is well-written in both standard and scientific English, well-organized, and its data are largely unproblematic; its original contribution to scholarly research is also evident. I do not have any concerns about its equations or statistical explanations.
Reply: Thank you for your affirmation. Your affirmation is an important supporting factor for the publication of this paper, so I would like to express my heartfelt gratitude to you.

In the real world, organized cyberattacks are rarely categoric or unidimensional, often integrating approaches by disdaining loss, following the path of least resistance and concentrating efforts. I wonder if the Author thinks it is pertinent to include a more detailed comparison of how the AKS mechanism is mobilized against multidimensional attacks, since this would be the best representation of feasibility and the project is designed in-itself for performance.
Reply: Thank you for your suggestions. According to your advice, the content at the end of the results section in this paper has been revised, which discusses how the AKS mechanism mobilizes against multi-dimensional attacks, thereby improving the practicality and value of this study. In real-world multi-dimensional attack scenarios, the AKS mechanism achieves targeted mobilization by dynamically perceiving data distributions and attack features. When it is detected that mixed attacks cause the data skewness to exceed the threshold θ=1.5, the mechanism automatically triggers the Sigmoid kernel to handle sparse boundaries, while adjusting the kernel parameter weights according to the attack perception factor $\alpha_{c}$ to strengthen the decision boundaries of high-risk attack dimensions.

In real-world applications, the SVM property of “insufficient processing capability of high-dimensional nonlinear data” poses a problem for advanced computation requirements. How does the Author propose to circumvent clear obstacles to implementation? For example, would parameter adjustment, parameter refinement and kernel function switching be feasible simultaneous yet separate processes at larger scales for consumers? I propose the Author include some text about implementation potential, especially since past reviewers seemed primarily skeptical due to an inherent supposition of novel computation leading to restricted implementation.
Reply: Thank you for the questions raised. According to the above questions, the discussion section has been revised to provide a discursive response, thereby enhancing the rationality of this paper and highlighting the implementation potential of this study. In practical applications, aiming at the capability bottleneck of SVM in processing high-dimensional non-linear data, the authors propose a triple optimization mechanism: adopting a lightweight parameter update strategy based on a 500-sample sliding window to reduce the computational load of kernel parameter adjustment; designing a parallel kernel function switching architecture to achieve independent calculation and result fusion of RBF and Sigmoid kernels through CPU multi-threading; introducing a progressive feature dimensionality reduction scheme, which first retains principal components with 95% variance through PCA, and then combines dynamic kernel adjustment. These mechanisms enable the model to maintain high detection accuracy and low memory occupation on edge computing devices, effectively avoiding high-dimensional computational bottlenecks and verifying the feasibility of large-scale deployment. Parameter adjustment, refinement, and kernel function switching can be carried out independently simultaneously to improve the model's adaptability and efficiency.

In terms of capital influx some form of concomitance or proposed simultaneity is certain, being that cyberthreat acting which is not directly strategic in terms of impact (DDoS, Ransomware) is nevertheless perpetrated in real-time (Phishing). However, Figure 7 likely also represents user response to critical frontend impacts of cyberthreat intrusion or intrusion detection since resource-intensive data collection, response and prevention processes as well as backend measures drive exponential bottleneck changes to user experience causing a greater number of attempted logins, varying user response to load times, etc. Should some mention of this be made in the limitations, in the form of identifying that trade-offs may be certain?

Reply: Thank you for your reminder. According to your reminder, the content of the conclusion section has been revised, in which the limitations you mentioned are discussed and analyzed to improve the rationality and quality of this paper. In terms of limitations, the study does not fully explore the impact of the concomitance between capital inflow and real-time attacks (such as phishing) on user experience—resource-intensive security measures may cause front-end bottlenecks such as loading delays, leading to a surge in user login attempts and changes in behavior patterns. In addition, the computational resource overhead of the model in the scenario of ultra-large-scale real-time data streams (such as more than 100,000 samples per second), such as the CPU occupancy rate of dynamic kernel function switching, still needs further optimization. Future research can combine user experience quantitative indicators (such as loading time and operation success rate) to build a multi-objective optimization model of "security protection-user perception", and introduce online learning algorithms to improve the adaptability of edge computing devices, so as to perfect the technology-behavior collaboration framework for network security governance.

Reviewer #3: 1. In order to improve clarity and focus, consider a revised version title like “Enhanced SVM-Based Model for Predicting Cyberspace Vulnerabilities: Analyzing the Role of User Group Dynamics and Capital Influx”
Reply: Thank you for your comments. According to your suggestions, the title of this paper has been revised to “Enhanced SVM-Based Model for Predicting Cyberspace Vulnerabilities: Analyzing the Role of User Group Dynamics and Capital Influx”.

2. Some sentences are dense and hard to follow (e.g., “by integrating the dynamics of user behavior change with the logic of platform capital expansion”). Try to simplify language and use more direct phrasing without diluting the academic tone.
Reply: Thank you for your suggestions. The sentences throughout the paper have been optimized according to your advice, so as to improve the overall quality of the content.

3. Terms like “prediction efficiency” and “platform capital expansion” may be vague or open to multiple interpretations. Thus, replace these with more conventional terms like computational efficiency or platform scaling due to investment.
Reply: Thank you for your suggestions. According to your suggestions, some professional terms in this paper have been processed to improve their readability and rationality.

4. Eliminate redundancy, especially in the description of TASVM's innovation. Avoid restating the same contributions multiple times.

Reply: Thank you for your reminder. The introduction section has been revised according to your reminder to reduce the redundancy in discussing the contributions of this paper. This study proposes a Threat-Aware Support Vector Machine (TASVM) model, which enhances detection capabilities through three innovative mechanisms: ① a dynamic kernel parameter adjustment mechanism based on data distribution, which real-time couples attack perception factors with data statistical features; ② a kernel parameter optimization mechanism introducing attack type risk factors to achieve differentiated weight allocation for different attack scenarios; ③ an intelligent kernel function switching strategy based on data skewness thresholds, which adaptively matches kernel functions to handle different distribution characteristics. These mechanisms collaboratively enhance the model's non-linear representation ability and risk sensitivity.

Reviewer #4: - Comparative analysis of literature review is missing need to add along with their results readings
Reply: Thank you for your suggestions. The literature review section has been revised according to your suggestions to conduct a comparative analysis of the results. Cybersecurity has become a core issue in the field of information technology. With the complexity and diversification of network attack methods, the importance of vulnerability prediction technology has become increasingly prominent. For example, Almaiah et al. (2022) analyzed common network attack types such as DDoS and malware, pointing out that traditional rule-based vulnerability detection methods can no longer cope with the current complex attack patterns, and their miss detection rate in new hybrid attack scenarios is as high as 32%. The machine learning-based intrusion detection system proposed by Samanta et al. (2021) improves the recognition rate of conventional attacks to 89% through historical data feature learning, but the F1-score drops sharply to 72% when processing non-linear data.

- In the discussion and conclusion section, no numerical results reading was discussed.
Reply: Thank you for your comments. According to your suggestions, the content of the discussion section in this paper has been revised, in which a more in-depth discussion and analysis of the result data of this paper are carried out, so as to improve the quality of the discussion in this paper. From the perspective of engineering deployment feasibility analysis, in the edge computing verification of the UNSW-NB15 dataset, the model shows the characteristics of low resource occupation (CPU utilization <20%, memory consumption <384MB) and strong cross-platform adaptability. Through the Docker containerization deployment scheme, it has completed the real-time vulnerability detection verification in the Ubuntu 20.04 and Windows Server 2019 environments, and the interface compatibility rate with mainstream intrusion detection systems such as Suricata reaches 92%. User behavior analysis further reveals that when the platform load exceeds the threshold (concurrent connections >5000), the correlation coefficient between the attack frequency and user defense behavior reaches 0.72 (p<0.01). The model provides a quantitative basis for dynamic adjustment of security strategies by capturing the causal chain of "capital inflow - user growth - attack exposure" (for example, the incidence of DDoS attacks increases by 37% during the peak period of platform financing).

- when dealing with attack risk not seen any category of attack basis on severity or risk of attack to deal woth it on priority.
Reply: Thank you for your comments. According to your suggestions, the content of the discussion section in this paper has been revised, which includes the discussion on the attack classification based on attack severity or risk, so as to improve the rationality of this paper. In the attack risk treatment, the research constructs a linkage mechanism between the severity-based attack classification system and indicators of compromise to optimize risk disposal. First, according to the dimensions of potential attack impact, propagation speed, and destructive capability, attacks are divided into three levels: high-risk (such as ransomware, DDoS attacks), medium-risk (such as phishing attacks, vulnerability exploitation), and low-risk (such as port scanning, information gathering). High-risk attacks can lead to system paralysis or data loss and require prioritized allocation of defense resources; medium-risk attacks may cause data breaches and need real-time monitoring; low-risk attacks have less harm and can be disposed of regularly.

- indicator of compromise role in the attack detection and risk of attack is totally neglected in the said study, can be a major factor and need to discussed and find from true positive attacks.

Reply: Thank you for your guidance. In the discussion section, the role of Indicators of Compromise (IOC) in attack detection and attack risk has been discussed as per your instructions. IOCs are introduced as key evidence for attack classification, with real-time acquisition of malicious IPs, hash values, C2 domain names and other information through threat intelligence platforms. For example, when detecting characteristic IOCs of ransomware (such as the hash value of the encryption algorithm of known ransomware or the C2 communication domain name), the system automatically classifies it as a high-risk attack and triggers an emergency response process; if IOCs of phishing attacks are found (such as counterfeit domain names or phishing email characteristics), it is classified as a medium-risk attack and the interception mechanism is activated. By constructing a three-dimensional classification model of "attack characteristics - risk level - IOC evidence", automatic priority ranking of attacks is realized. Combined with the NVD risk scoring system, dynamic weights are assigned to attacks of different levels to improve the identification efficiency and response speed of high-risk attacks, forming a more accurate risk disposal framework.

6. PLOS authors have the option to publish the peer review history of their article ([what does this mean?](https://journals.plos.org/plosone/s/editorial-and-peer-review-process#loc-peer-review-history)). If published, this will include your full peer review and any attached files.


If you choose “no”, your identity will remain anonymous but your review may still be made public.


**Do you want your identity to be public for this peer review?** For information about this choice, including consent withdrawal, please see our [Privacy Policy](https://www.plos.org/privacy-policy).

Reviewer #1: No

Reviewer #2: **Yes:** Paul-André Betito, HBA*, MSW, RSW

Reviewer #3: No

Reviewer #4: No
